# Prevalence of refractive error in Portugal estimated from ophthalmic lens manufacturing data: Ten-years analysis

**DOI:** 10.1371/journal.pone.0284703

**Published:** 2023-04-21

**Authors:** Vera L. Alves Carneiro, José Manuel González-Méijome

**Affiliations:** Clinical and Experimental Optometry Research Lab (CEORLab), Center of Physics (Optometry), School of Sciences, University of Minho, Braga, Portugal; University of Ulster, UNITED KINGDOM

## Abstract

**Purpose:**

To investigate the prevalence, distribution and trends of refractive error from ophthalmic lens manufacturing data over a ten-year period.

**Methods:**

Fully anonymized data from ophthalmic lenses, for the years between 2010 and 2020, provided by the leading ophthalmic lens manufacturer operating in Portugal were analysed (no human participants were involved in the research). Prescriptions delivered were divided in single vision prescriptions and progressive/multifocal prescriptions and categorized into 14 spherical equivalent ranges. Given the lack of absolute values, indirect estimates and a qualitative analysis of the current situation and trends on refractive error epidemiology was carried out.

**Results:**

Dataset from manufacturer comprises percentage values of ophthalmic lenses dispensed in Portugal. The distribution of ophthalmic prescriptions for single vision prescriptions presents most of the observations in the range [-1.49, -0.50] diopters, in every year from 2010 to 2020. For the progressive prescription’s lenses, most of the observations is in an interval of two ranges, [0.50, 1.49] and [1.50, 2.99] diopters. From 2010 to 2020 the proportion of single vision ophthalmic lens prescriptions for myopia increased from 38.13% to 46.21%; the proportion for high myopia increased from 2.76% to 4.45%; and the proportion for hyperopia decreased from 40.85% to 31.36%.

**Conclusions:**

Ophthalmic lens manufacturing data can be a valuable source for long-term analysis of refractive error prescription and trends over time. It was possible to observe a trend of increasing prevalence of myopia and high myopia from 40.89% in 2010 to 50.66% in 2020. That increase trend has important implications for public health and in the planning of services.

## Introduction

Refractive errors are considered a public health challenge affecting all age groups and with important implications on the individual development and quality of life and on society economics and productivity[1–4]. Due to the importance of epidemiological data on refractive error, this is a research area of continuing interest.

To investigate the prevalence of refractive error, their trend and progression, and to adopt a strategy for prevention and treatment of these conditions, should be a public health priority, in particular if noted that future generations may become even more myopic [5]. Recent European refractive error estimates have been derived from large epidemiological studies or meta-analysis [6]. Results showed that the prevalence of myopia has increased dramatically in just two generations from less than 15% in the cohorts aged 29–53 years old to over 45% in the younger cohorts aged under 29 years. A recent estimate of the myopia prevalence in Portugal based on a meta-analysis of different studies provided an estimate of 31.9% [7].

However, epidemiological studies and the subsequent systematic reviews and meta-analysis take huge amounts of resources, time and do not allow to keep updated estimates overtime. So, more accessible, less time and resource consuming data gathering is necessary to continuously monitor refractive error trends and eventually adjust eye care service schemes.

To do so, updated, valid and accessible refractive error epidemiological data must be available and are essential for planning more effective eye care services, to offer evidence-based interventions, and to allocate resources effectively. Ophthalmic lens manufacturer big data can, in the absence of population-based surveys, represent a potential source of refractive error epidemiological data, providing a fast and cost-effective substitute measure of refractive error distribution [8].

This is the first estimate of refractive error prescriptions in Portugal from data collected from ophthalmic lens manufacturer. This is also the first longitudinal estimate of changes in the patterns of prescriptions. Altogether, and despite the limitations of this methodology, the longitudinal analysis provides the opportunity to evaluate trends overtime and to use this information in the planning of refractive eye care services.

## Methods

Fully anonymized data from ophthalmic lenses, presented as a percentage of the total dispensed lenses in Portugal and not as absolute values, were provided by a leading ophthalmic lens manufacturer operating in Portugal. The data provided were categorized according to the spherical equivalent ranges.

Estimates from Portugal over the last 10 years show that about 50% of the population wear spectacles, which represents about 5 million persons. Moreover, about 8.9%, or 733.000 people over the age of 15 years old, are contact lens wearers [9]. According to an official report, the manufacturer in question represents approximately 55% of the national market of ophthalmic lenses [10], this means that the dataset represents about 2.5 to 3 million prescriptions per year. Assuming every patient updates the prescription on average every 3 years, this will represent a dataset of about 10 million prescriptions and 20 million individual lenses. This can be considered representative of the Portuguese population.

This dataset comprises ophthalmic lenses that were delivered in the Portuguese territory after a prescription made by an eye care practitioner. These data don’t allow to identify the methods of assessment of the refractive error or any other information about the ophthalmic lens user (sex, age, ethnicity or other).

Data were divided in two groups: single vision prescriptions and progressive/multifocal prescriptions. It included total percentage of ophthalmic lenses for each year categorized into 14 spherical equivalent ranges. Data were validated for missing or incomplete data fields. Given the lack of absolute values, indirect estimates and a qualitative analysis of the current situation and trends on ophthalmic prescriptions was carried out.

The quantitative definitions from the International Myopia Institute have been adopted, myopia was defined as spherical equivalent refractive error ≤ -0.50 Diopters when ocular accommodation is relaxed and high myopia as spherical equivalent refractive error ≤ -6.00 Diopters when ocular accommodation is relaxed [11]. Emmetropia was defined for those with a spherical equivalent of less than 0.50 Diopters in absolute value, regardless of whether the blur is myopic or hyperopic, and hyperopia when the spherical equivalent is ≥ +0.50 Diopters [12].

## Results

Dataset from manufacturer comprises percentage values of ophthalmic lenses dispensed in Portugal between the years of 2010 to 2020 divided in single vison prescriptions and progressive/multifocal prescriptions. Total percentage values were categorized into 14 spherical equivalent ranges for both groups single vison prescriptions (Table 1) and progressive/multifocal prescriptions (Table 2).

**Table 1:** Total percentage values according 14 spherical equivalent (SE) ranges for the non-progressive/single vison prescriptions group.

SE	≤ -20.00	[-19.99; -15]	[-14.99; -10]	[-9.99; -8.00]	[-7.99; -6.00]	[-5.99; -3,00]	[-2.99; -1.50]	[-1.49; -0,50]	[-0.49; -0.01]	0,00	[0.01;0.49]	[0.50; 1.49]	[1.50; 2.99]	[3.00; 5.99]	[6.00; 9.99]	≥10.00
**2010**	0.025%	0.114%	0.470%	0.611%	1.545%	7.928%	12.882%	17.322%	8.951%	3.401%	5.898%	12.869%	16.087%	10.884%	0.919%	0.096%
**2011**	0.030%	0.094%	0.462%	0.603%	1.471%	7.910%	13.515%	17.711%	8.988%	3.347%	5.532%	12.159%	16.241%	10.965%	0.889%	0.084%
**2012**	0.029%	0.095%	0.423%	0.557%	1.338%	7.396%	13.257%	18.459%	9.525%	3.473%	5.659%	12.129%	15.939%	10.781%	0.853%	0.086%
**2013**	0.030%	0.108%	0.468%	0.629%	1.486%	8.021%	13.383%	17.517%	8.581%	2.024%	5.656%	12.469%	16.629%	11.976%	0.991%	0.034%
**2014**	0.042%	0.129%	0.547%	0.700%	1.674%	8.345%	12.557%	15.649%	7.294%	3.535%	5.102%	12.027%	16.741%	14.324%	1.229%	0.104%
**2015**	0.046%	0.152%	0.622%	0.752%	1.832%	8.863%	12.891%	16.029%	7.431%	3.470%	5.007%	11.392%	15.842%	14.289%	1.280%	0.102%
**2016**	0.045%	0.154%	0.652%	0.828%	1.956%	9.396%	13.517%	16.494%	7.828%	3.670%	4.974%	10.641%	14.937%	13.562%	1.248%	0.100%
**2017**	0.053%	0.159%	0.664%	0.882%	2.076%	10.016%	14.042%	17.058%	8.195%	3.953%	5.145%	10.744%	14.346%	11.495%	1.060%	0.112%
**2018**	0.047%	0.128%	0.622%	0.804%	2.065%	10.888%	16.176%	18.853%	8.239%	5.835%	5.118%	10.419%	12.284%	7.741%	0.691%	0.091%
**2019**	0.030%	0.095%	0.518%	0.771%	2.013%	10.762%	16.332%	18.875%	8.664%	4.244%	5.356%	10.777%	12.534%	8.297%	0.660%	0.073%
**2020**	0.032%	0.149%	0.765%	1.011%	2.488%	11.744%	15.866%	18.597%	8.844%	4.017%	5.124%	10.261%	12.216%	8.031%	0.777%	0.079%

**Table 2:** Total percentage values according 14 spherical equivalent (SE) ranges for the progressive/multifocal prescriptions group.

SE	≤ -20.00	[-19.99; -15]	[-14.99; -10]	[-9.99; -8.00]	[-7.99; -6.00]	[-5.99; -3,00]	[-2.99; -1.50]	[-1.49; -0,50]	[-0.49; -0.01]	0,00	[0.01;0.49]	[0.50; 1.49]	[1.50; 2.99]	[3.00; 5.99]	[6.00; 9.99]	≥10.00
**2010**	0.000%	0.022%	0.197%	0.332%	0.815%	4.077%	5.887%	10.473%	7.163%	3.464%	8.530%	26.643%	25.811%	6.293%	0.265%	0.028%
**2011**	0.000%	0.014%	0.170%	0.299%	0.752%	3.938%	5.759%	10.484%	7.158%	3.455%	8.635%	26.783%	25.957%	6.349%	0.229%	0.019%
**2012**	0.001%	0.015%	0.160%	0.264%	0.732%	3.911%	5.788%	10.765%	7.321%	3.511%	8.421%	26.670%	25.891%	6.291%	0.245%	0.016%
**2013**	0.001%	0.017%	0.139%	0.273%	0.703%	3.801%	5.714%	10.811%	7.296%	3.483%	8.354%	26.511%	26.415%	6.221%	0.240%	0.022%
**2014**	0.003%	0.017%	0.163%	0.282%	0.693%	3.834%	5.776%	10.936%	7.353%	3.497%	8.310%	26.194%	26.246%	6.429%	0.253%	0.014%
**2015**	0.001%	0.015%	0.188%	0.288%	0.710%	3.842%	5.771%	10.976%	7.456%	3.856%	8.495%	25.877%	25.812%	6.431%	0.268%	0.015%
**2016**	0.001%	0.022%	0.170%	0.289%	0.787%	3.973%	5.754%	11.109%	7.730%	4.230%	8.607%	25.550%	25.174%	6.289%	0.299%	0.014%
**2017**	0.003%	0.024%	0.205%	0.315%	0.778%	3.936%	5.828%	11.204%	7.866%	4.314%	8.602%	25.257%	24.916%	6.421%	0.315%	0.016%
**2018**	0.001%	0.016%	0.130%	0.272%	0.848%	4.553%	6.541%	11.733%	7.959%	4.324%	8.539%	25.040%	23.950%	5.790%	0.289%	0.016%
**2019**	0.001%	0.025%	0.212%	0.293%	0.833%	4.397%	6.198%	11.602%	7.885%	4.164%	8.523%	24.769%	24.464%	6.311%	0.303%	0.018%
**2020**	0.002%	0.019%	0.190%	0.295%	0.804%	4.074%	5.768%	11.092%	7.953%	4.270%	8.545%	24.923%	25.174%	6.569%	0.310%	0.013%

**Fig 1:**
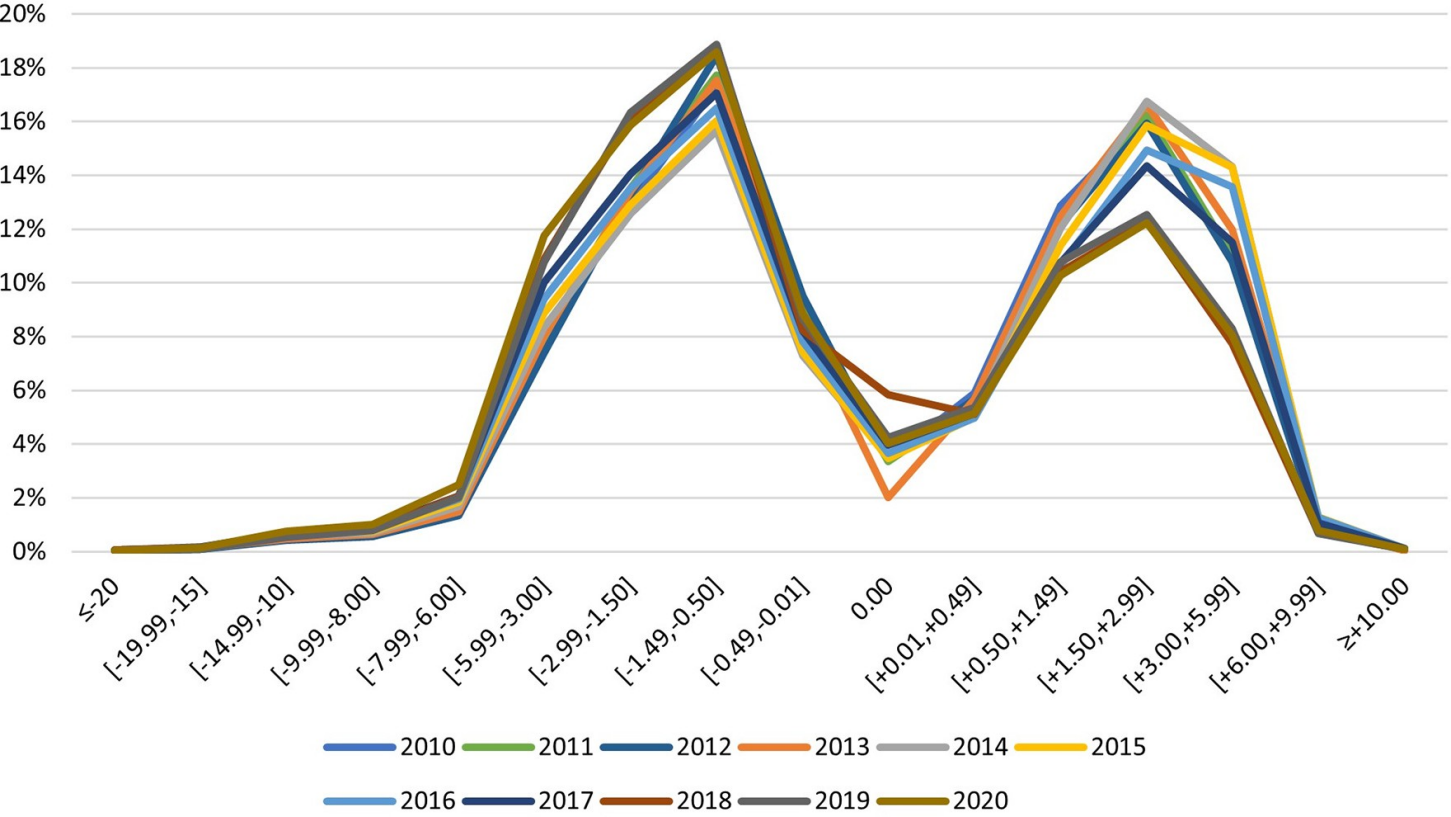
Distribution of ophthalmic prescriptions in Portugal from 2010 to 2020—single vision.

The distribution of ophthalmic prescriptions delivered in Portugal from 2010 to 2020, for single vision prescriptions, (Fig 1) resembles a bimodal curve with the majority of the observations in the range [-1.49, -0.50], in every year from 2010 to 2020.

**Fig 2:**
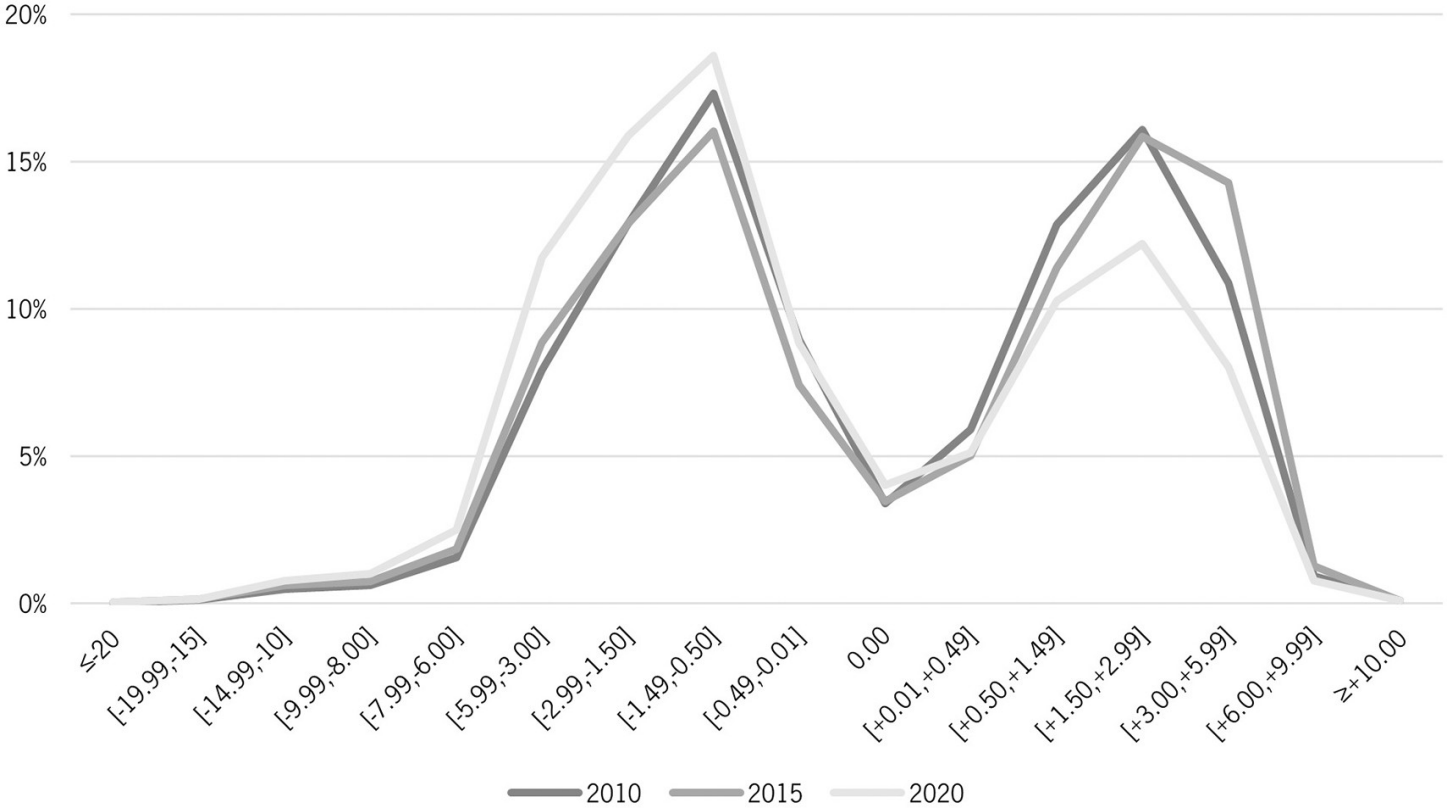
Distribution of ophthalmic prescriptions in Portugal in 2010, 2015 and 2020—single vision.

The same graphic representation of the ophthalmic prescription distribution, but focusing only on the chosen cut-off years of 2010, 2015 and 2020 was made to better observe variations in the distribution over the decade (Fig 2).

**Fig 3:**
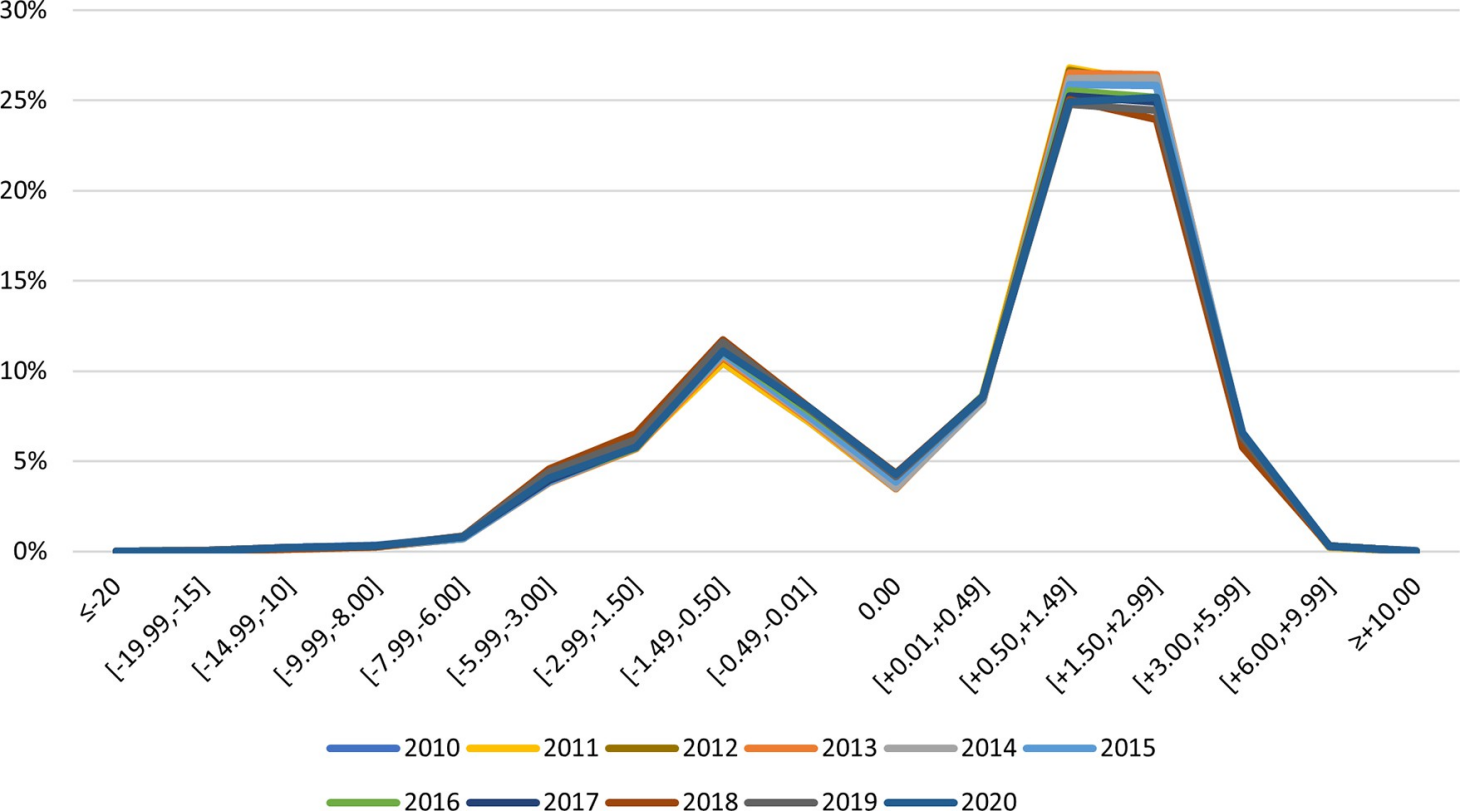
Distribution of ophthalmic prescriptions in Portugal from 2010 to 2020—progressive.

For the progressive prescription’s lenses, the distribution of ophthalmic prescriptions in Portugal for the same years, presents most of the observations in an interval of two ranges, [0.50, 1.49] and [1.50, 2.99], (Fig 3). Interestingly, these two ranges reduced their representativeness between from 52.45% in 2010 to 50.10% in 2020.

**Fig 4:**
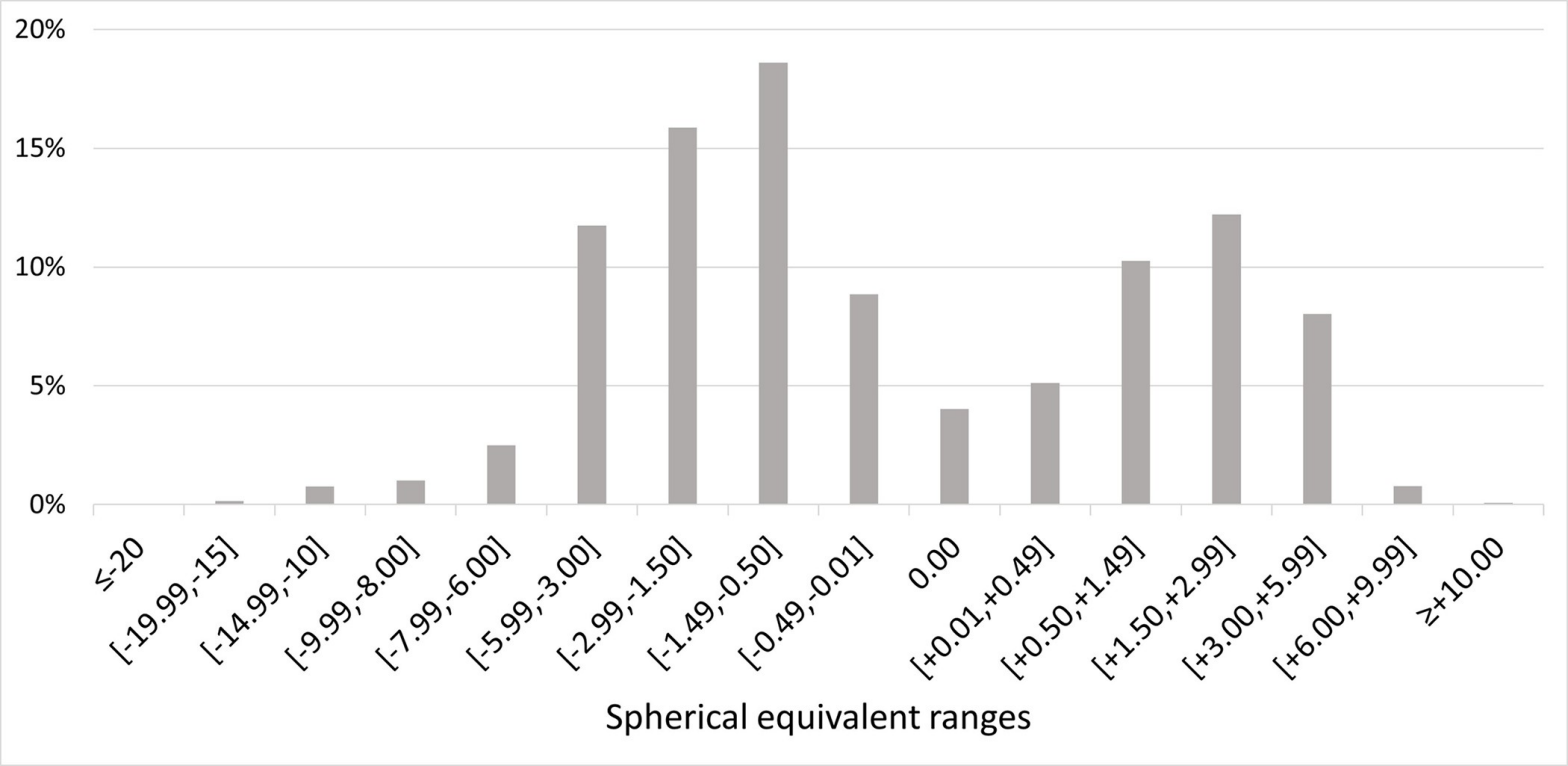
Distribution of ophthalmic prescriptions in Portugal in 2020 according to single vision ophthalmic lenses prescriptions. Dashed line shows average fitting to data.

For an analysis of the refractive error distribution in 2020, and given the origin and characteristics of the data, only the data set related to the single vision prescriptions were used (Fig 4).

For a trend analysis from 2010 to 2020, and given the origin and characteristics of the data, only the data set related to the non-progressive/single vision prescriptions were used.

Grouped percentages for each refractive error, high myopia, myopia, emmetropia and hyperopia, as previously defined, were obtained by the sum of each spherical equivalent ranges for the years of 2010 and 2020 (Table 3). On average, the percentage of all myopic non-progressive/single vision prescriptions increased by nearly 1%/year from 40.90% in 2010 to 49.40% in 2019 before Covid pandemic, and to 50.65% in 2020. High myopia also increased during these periods.

**Table 3:** Percentage of refractive error type in the first and last years of measurement.

	High Myopia	Myopia (All)	Emmetropia	Hyperopia
**2010**	2.77%	40.90%	18.25%	40.86%
**2019 (pre-COVID)**	3.43%	49.40%	18.26%	32.34%
**2020 (COVID)**	4.45%	50.65%	17.99%	31.36%

**Fig 5:**
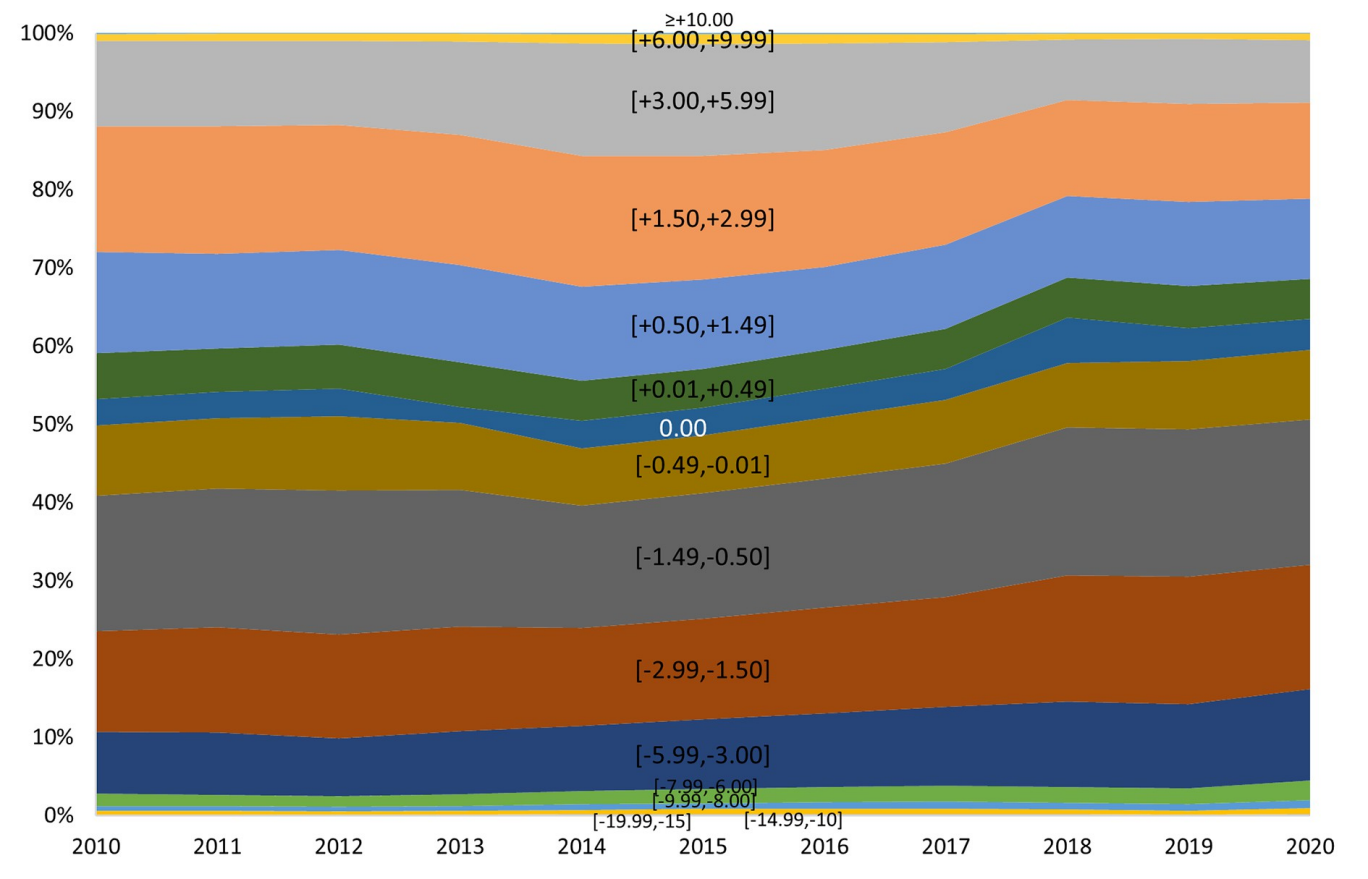
Trends in the distribution of ophthalmic prescriptions in Portugal from 2010 to 2020 according to single vision ophthalmic lenses prescriptions.

A graphical representation was made to analyse the trends in the distribution of ophthalmic prescriptions in Portugal from each year, since 2010 to 2020 (Fig 5).

**Fig 6:**
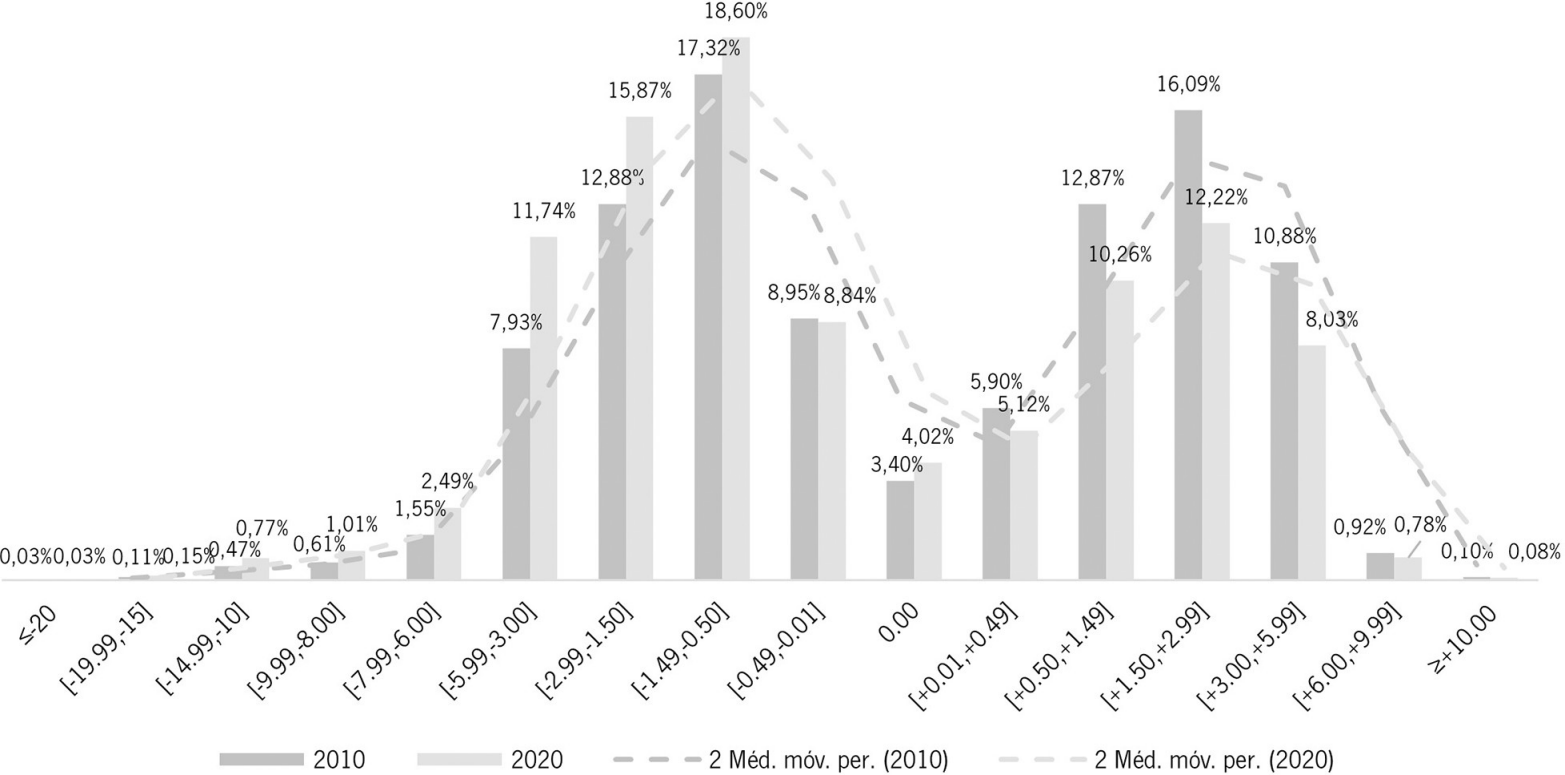
Trends in the distribution of ophthalmic prescriptions in Portugal for the years of 2010 and 2020 for each spherical equivalent range according to single vision ophthalmic lenses prescriptions. Dashed lines show average fitting and 2010–2020 shift in prescription delivery patterns.

A graphical representation was made to compare each one of the spherical equivalent ranges in the year of 2010 and the year of 2020 to analyse differences and assess trends between the two extremes years of the decade (Fig 6).

## Discussion

This study approaches an alternative method to estimate refractive error distribution through ophthalmic lens manufacturer big data analysis. In the absence of population-based surveys, this approach can represent a potential source of refractive error epidemiological data [8].

However, some important limitations need to be acknowledged. First, ophthalmic lenses are not the only form available for refractive error correction. Alternative corrections such as contact lenses or refractive surgery are becoming increasingly popular. However, ophthalmic lenses are undoubtedly the most common intervention for refractive error correction and most contact lens wearers also use ophthalmic lens [13]. Secondly, when using data from devices only the people with access to them are considered, which leaves everyone with an uncorrected refractive error out of the equation. Thirdly, the nature of the data acquired does not allow conclusions about distribution by sex, age groups or other subpopulation divisions. And lastly, the method of assessment of the refraction is impossible with this methodology. Despite that, the very large sample size that ophthalmic lens manufacturer big data represents, makes it unlikely that all these limitations have significant impact on the results, particularly when compared on a longitudinal perspective. The inclusion of data from an atypical year as 2020, under SARS-Covid pandemic is an additional concern. However, the authors did not find any anomalous trends that justify the removal of these data. So, for the sake of integrity, we decided to keep this data. In this context is worth saying that in Portugal, optometric services and optical shops remained open as primary care setups during most of the pandemic time, so the prescription and distribution of optical appliances was minimally impacted. The stability of the data might suggest that, but this study does not intend to derive information about the ophthalmic prescription during pandemic time.

These data do not account for contact lens wearers or refractive surgery patients. Though there is no official public data on the penetration of these prescriptions, they should have represented a much lower percentage compared to spectacle prescriptions. There other limitations related with the fact that astigmatic prescriptions are converted into spherical equivalent and a 0.75 cylinder prescription will be counted in the emmetropic group. Emmetropia is of course underrepresented in this data as all emmetropes who do not need a prescription are not represented here.

Despite all these limitations, and the level of uncertainty they introduce, we consider that the value of this data is in the comparative perspective over the years as a methodology to provide ready accessible relevant information for evaluating trends in refractive error in the population, rather than providing absolute values of prevalence representative of the whole population.

It is also important to note that, the classic distribution curve found in refractive error studies, with most observations centred near the emmetropia [14] would never be possible to observe in studies using big data from manufacturers. Data from manufacturers refer to an optical correction device prescribed assuming a certain refractive error which disregards practically all emmetropic individuals. This fact can be observed by the atypical distribution of ophthalmic prescriptions in Figs 1–3.

According to the report Vision Needs Monitor EMEA 2019, with a sample in Portugal of 0,05% of the total population in the country aged 15 years or over, close to 80% of the respondents at some point in their life needed ophthalmic lenses and almost 70% currently use them. Knowing that the manufacturer that has provided this data holds a very relevant market position in Portugal, approximately 55%, we can assume that this dataset is very close to the reality of ophthalmic lens users in Portugal.

The distribution of ophthalmic prescriptions in Portugal from 2010 to 2020 for non-progressive/single vision prescriptions (Fig 1) resembles a bimodal curve with the majority of the observations in the range [-1.49, -0.50] and more oriented to the myopic prescriptions. For the non-progressive/single vision lenses, in every year from 2010 to 2020 is possible to observe a higher percentage of myopia prescriptions in relation to hyperopia, and from 2018 onwards the percentage of myopia and high myopia represent 50% of the total prescriptions made for single vision, in line with what is happening in most developed countries and in long-term forecasts [5,15,16]. The general trend observed—a higher percentage of myopia prescriptions in relation to hyperopia over the decade—is also observed in 2020 even with the limitations in the access to the health systems caused by the pandemic situation [17].

Comparing the distribution of ophthalmic prescriptions in Portugal only between the two extreme years of the decade, 2010 and 2020 (Fig 3), we can observe a shift to a more myopic population over the decade, with an increase of all the myopic spherical equivalent intervals and a decrease of all hyperopic ones. The spherical equivalent range of [-5.99, -3.00] was the one that increased the most, by additional 2.984% from 2010 to 2020 and the spherical equivalent range of [1.50, 2.99] was the one that decreased the most, with a reduction of 3.871% from 2010 to 2020.

In the case of the progressive/multifocal prescriptions distribution (Fig 2), the majority of the observations are noted in an interval of two ranges, [0.50, 1.49] and [1.50, 2.99]. A higher percentage of hyperopic prescriptions is observed but is important to note a gradual increase in myopic prescriptions over the decade and a decrease in the same order of the hyperopic. Considering that these data refer to presbyopic individuals, usually in an age group above 45 years old, we can infer, supported by recent scientific evidence [18–20], that the new presbyopic persons that have emerged in recent years have a more myopic trend than the previous ones.

For analysing the epidemiology of refractive error in 2020 and the trends from 2010 to 2020 only the data set related to the non-progressive/single vison prescriptions were used in order to not consider presbyopia, an age-related condition that mostly affects individuals over 45 years of age.

In 2020 (Fig 4), 50.66% of the prescriptions made were for myopia and high myopia correction. In the same year, 17.98% of prescriptions were for emmetropia and 31.36% for hyperopia (Table 3). These data are representative of the total burden of myopia among the other refractive error and are in line with what was observed in other countries and extrapolated for Portugal [5,6,21,22].

More concerning is the comparative analysis of the burden of each refractive error in ophthalmic lens prescriptions for the extreme years of the decade, 2010 and 2020. From 2010 to 2020 the proportion of myopia increased from 38.13% to 46.21%; the proportion of high myopia increased from 2.76% to 4.45%; and the proportion of hyperopia decreased from 40.85% to 31.36% (Table 3). Holden *et al*., 2016 [16], estimated an increase in global myopia prevalence from 28.3% in 2010 to 33.9% in 2020, this work, with the limitations inherent to the data characteristics, allows to observe an increase in myopia from 38.13% in 2010 to 46.21% in 2020, that, in terms of the percentage increment value, is very close to the predictions. This helps to support the case that manufacturer big data are a timely and cost-effective alternative data source for monitoring the distribution of refractive error. These trends in the increase of high myopia and myopia and decrease of emmetropia and hyperopia prevalence were also observed in a cross-section prospective study that analysed records from patients in Portugal in 2021 (submitted for publication) and by Jorge *et al*, 2016, in 12 years study among student population [23].

It is possible to observe over the course of the decade (Figs 5 and 6) a trend of gradual and significant increase in myopia and high myopia, which allows to anticipate the impact on public health. Trends of refractive error in Portugal in the last decade are very similar to the observed in most developed countries [16], and myopia, high myopia included, represents in 2020 more than a half the total prescriptions made for single vision by this manufacturer. Refractive error, specifically myopia, when uncorrected or undercorrected can affect the educational development, school performance, and limit future opportunities of employability and quality of life. Additionally, myopia-related ocular complications include cataract, retinal detachment, choroidal/scleral thinning, myopic choroidal neovascularization, glaucoma, among others with high probability to cause visual impairment [24]. The burden of myopia, knowing its progression and impact, must therefore be addressed from a public health perspective, with universal and effective coverage [13,25].

Assuming that in the future, even nations that have little myopia today will be severely affected [16], early detection to avoid impact on the individual’s development [26], as well as the adoption of preventive mechanisms for risk factors [27,28] and slowing progression [29], are measures that these data show an urgent need to adopt by the Portuguese authorities. Of particular concern is the increase in high myopia. Although values are low, this data showed that from 2010 to 2020 there has been a nearly 2-fold increase in high myopes that can be at risk of developing pathology, and consequently could add to the burden of vision impairment 10–20 years in the future [30].

## Conclusions

Despite the known individual variations in the prevalence of myopia and high myopia, according to geography, age or ethnic groups, that are impossible to estimate with the data from this study, it is possible to observe a trend of increasing percentage of myopia and high myopia from 40.90% in 2010 to 50.65% in 2020. That increase trend has important implications for public health, in the planning of services, indicating the need to adopt promotion, prevention, treatment and rehabilitation eye care services to manage not only the myopia, but also, myopia-related complications likely to cause visual impairment. Additionally, this study allows to identify alternative sources of epidemiological data, such as manufacturers big data, and places this data as an important instrument for public health purposes.

## Supporting information

S1 File Dataset from manufacturer that comprises percentage values of ophthalmic lenses dispensed in Portugal between the years of 2010 to 2020. (XLSX)
